# Test-Retest Reliability and Physiological Responses Associated with the Steep Ramp Anaerobic Test in Patients with COPD

**DOI:** 10.1155/2012/653831

**Published:** 2012-06-06

**Authors:** Robyn L. Chura, Darcy D. Marciniuk, Ron Clemens, Scotty J. Butcher

**Affiliations:** ^1^School of Physical Therapy, University of Saskatchewan, 1121 College Dr, Saskatoon, SK, Canada S7N 0W3; ^2^Respirology, Critical Care and Sleep Medicine, University of Saskatchewan, Canada

## Abstract

The Steep Ramp Anaerobic Test (SRAT) was developed as a clinical test of anaerobic leg muscle function for use in determining anaerobic power and in prescribing high-intensity interval exercise in patients with chronic heart failure and Chronic Obstructive Pulmonary Disease (COPD); however, neither the test-retest reliability nor the physiological qualities of this test have been reported. We therefore, assessed test-retest reliability of the SRAT and the physiological characteristics associated with the test in patients with COPD. 11 COPD patients (mean FEV_1_ 43% predicted) performed a cardiopulmonary exercise test (CPET) on Day 1, and an SRAT and a 30-second Wingate anaerobic test (WAT) on each of Days 2 and 3. The SRAT showed a high degree of test-retest reliability (ICC = 0.99; CV = 3.8%, and bias 4.5 W, error −15.3–24.4 W). Power output on the SRAT was 157 W compared to 66 W on the CPET and 231 W on the WAT. Despite the differences in workload, patients exhibited similar metabolic and ventilatory responses between the three tests. Measures of ventilatory constraint correlated more strongly with the CPET than the WAT; however, physiological variables correlated more strongly with the WAT. The SRAT is a highly reliable test that better reflects physiological performance on a WAT power test despite a similar level of ventilatory constraint compared to CPET.

## 1. Introduction

Individuals with Chronic Obstructive Pulmonary Disease (COPD) are often prescribed aerobic exercise to enhance function and reduce shortness of breath during activities of daily living. General guidelines for this exercise prescription suggest patients should exercise continuously at moderate intensities [1–3]. There is evidence, however, to suggest that exercise at higher intensities may be more beneficial for this population [4].

Traditionally, results from cardiopulmonary exercise testing (CPET) involving an incremental, graded exercise test (GXT) of 8–12 minutes in duration, have been used to prescribe exercise for individuals with COPD and are widely considered to be the gold standard for measurement of cardiopulmonary function and aerobic performance [5]. CPET, however, may underestimate the workload required for optimal physiological benefit from exercise training due to ventilatory limitations causing early test cessation and a blunted peak work rate [6, 7]. High-intensity interval exercise intensity may be prescribed for healthy individuals based on tests of anaerobic power and capacity, such as a 30-second Wingate Anaerobic Test (WAT), which is considered to be the gold standard measure of anaerobic capacity [8]; however, these types of tests have not been widely used, nor would be appropriate in typical clinical use for individuals with COPD. However, the steep ramp anaerobic test (SRAT) has been proposed as a clinical test that may more accurately reflect leg muscle capabilities and better set interval training intensities for individuals with chronic heart failure [7, 9, 10] and COPD [11].

The SRAT was developed by Meyer et al. [7] for use by patients with heart failure to specifically challenge the muscles maximally before patients reached a cardiovascular limit. Unlike the WAT, in which subjects must pedal as fast as possible against a fixed resistance for 30 seconds, the SRAT is an incremental GXT where the workload increases by 25 watts every 10 seconds until patient exhaustion [7, 10]. Much higher work rates are typically achieved with the SRAT compared to the incremental CPET, and a percentage of the peak work rate (PWR) from the SRAT can be used to prescribe intervals for training in this population [10]. The SRAT has also been used in COPD patients to prescribe intensity for high intensity interval exercise [11]. The SRAT may be better tolerated for use in populations that become short of breath quickly during exercise because, and rather than being a timed test like the WAT, it is patient-limited. The test-retest reliability and the physiological responses of the SRAT in this population remain unknown.

The purposes of this study were to determine (a) the test-retest reliability of the SRAT in patients with COPD and (b) the physiologic, ventilatory, and perceptual parameters obtained on the SRAT compared with performance on a traditional CPET or WAT in COPD patients.

## 2. Materials and Methods

### 2.1. Subjects

11 patients (7 males and 4 females) with moderate and severe COPD (11) were recruited through the Saskatoon Pulmonary Rehabilitation Program and through the Division of Respirology, Critical Care and Sleep Medicine, University of Saskatchewan. Subjects had a respirologist confirmed diagnosis of COPD [12], did not require the use of supplemental oxygen at rest or during exercise, and had not been in hospital with an acute exacerbation within the previous 6 weeks. Subjects were excluded if they had cardiovascular or musculoskeletal disease that would prevent them from completing heavy exercise.

This research was approved by the University of Saskatchewan Biomedical Ethics Committee. All subjects signed a consent form and were advised that they could freely withdraw from the study at any time.

### 2.2. Research Design

A randomized cross-over design was used to assess subjects' physiological, ventilatory, and perceptual responses to the SRAT as compared to the CPET and WAT. The subjects attended 3 sessions for testing, within a 3 week period, with at least 48 hours separating sessions. An initial baseline assessment session included screening, assessment of criteria for study admission, pulmonary function tests, and an incremental CPET. The following 2 visits each included a 30-second WAT and a SR test separated by one hour. The second of these 2 visits was included in order to establish the test-retest reliability of these measures. The order of the tests was constant between visits but randomized between subjects.

### 2.3. Pulmonary Function Testing and CPET

Resting pulmonary function testing (FEV_1_, FVC, RV, TLC, D_L_CO) was performed according to established standards [13] (V6200C Autobox and *V*_max⁡_ 229D gas analyzer, SensorMedics Corp., Yorba Linda, California, USA). CPET was performed using established protocols [5] with a workrate increment of 5–15 W/min on a mechanically braked cycle ergometer (800 S, SensorMedics). The test was terminated when the subject indicated voluntary exhaustion, or the revolutions per minute fell below 60 and could not be increased with encouragement. Peak work rate (CPET_peak_), and all physiologic, ventilatory, and perceptual measures were collected and used in the analysis.

### 2.4. 30-Second Wingate Anaerobic Test (WAT)

The WAT was performed as per established protocol [8]. Subjects completed a self-paced 5 minute warm-up on the cycle ergometer (Monark 894 E, Ergomedic). Subjects were given two practice trials where they were asked to pedal as fast as possible, and one half the brake weight used for the actual WAT was applied to the flywheel for two seconds. This protocol was repeated for a second practice trial. After a two minute rest, the WAT was performed. Patients were instructed to maintain the maximal velocity for 30 seconds against the full break weight (females: 35 g/kg [14] and males: 45 g/kg [15]). Continual standardized encouragement was given to the patient throughout the entire test. The average power output (WAT_avg_) over the 30 seconds (which reflects anaerobic capacity), and all physiologic, ventilatory, and perceptual measures were collected and used in the analysis.

### 2.5. The Steep Ramp Anaerobic Test (SRAT)

The SRAT was performed as described by Meyer et al. [7]. Testing was performed using the same equipment, with monitoring of the same parameters as for the CPET and WAT. After a 2 minute unloaded warm-up, the intensity increased by 25 watts every 10 seconds. The test was terminated when the subject indicated they could no longer continue or if the revolutions per minute fell below 60 rpm. Continual standardized encouragement was given to the patient throughout the entire test. The peak work rate (SRAT_peak_), and all physiologic, ventilatory, and perceptual measures were collected and used in the analysis.

### 2.6. Physiologic, Ventilatory, and Perceptual Measures

For all three exercise tests, physiologic measurements (blood pressure, heart rate (HR) and rhythm (3-lead ECG), oxygen saturation (SpO_2_) (N-395, Nellcor)), and perceptual measures (ratings of perceived exertion (RPE) for dyspnea and fatigue (0–10 modified Borg scale)), were obtained at baseline, during exercise, and end-exercise. Measurements including oxygen consumption (*V*_O_2__), carbon dioxide production (*V*_CO_2__), tidal volume (*V*_*T*_), minute ventilation (*V*_*E*_), and respiratory rate (RR) were recorded on a breath-by-breath basis and were averaged in 10 second increments. Inspiratory capacity (IC) maneuvers [16] were performed at baseline, during exercise, and end-exercise. From these maneuvers, operational lung volumes (end-expiratory lung volume (EELV) and end-inspiratory lung volume (EILV)) were calculated at each time point. EELV was estimated as the difference between TLC and IC, whereas EILV was estimated as the EELV plus *V*_*T*_. The degree of ventilatory constraint at peak exercise was evaluated by the inspiratory reserve volume (IRV; equals TLC−EILV) and by the *V*_*T*_/IC ratio.

### 2.7. Statistical Analysis

Test-retest reliability of the SRAT and the WAT was analyzed using Intraclass correlations (ICC), coefficient of variation (CV), and Bland-Altman plots. The analysis of the data comparing the CPET_peak_, SRAT_peak_, and the W_avg_, as well as the ventilatory, physiological, and perceptual measures for each of the three tests included repeated measures analysis of variance (ANOVA). Tukey's post hoc analysis was performed where significant differences were found. Pearson *r* correlations for the work rate, ventilatory, physiological, and perceptual measures of each of the three tests were also performed to determine significant relationships between measures. All statistical analyses were performed using a significance level of *P* < 0.05.

## 3. Results

**Figure 1:**
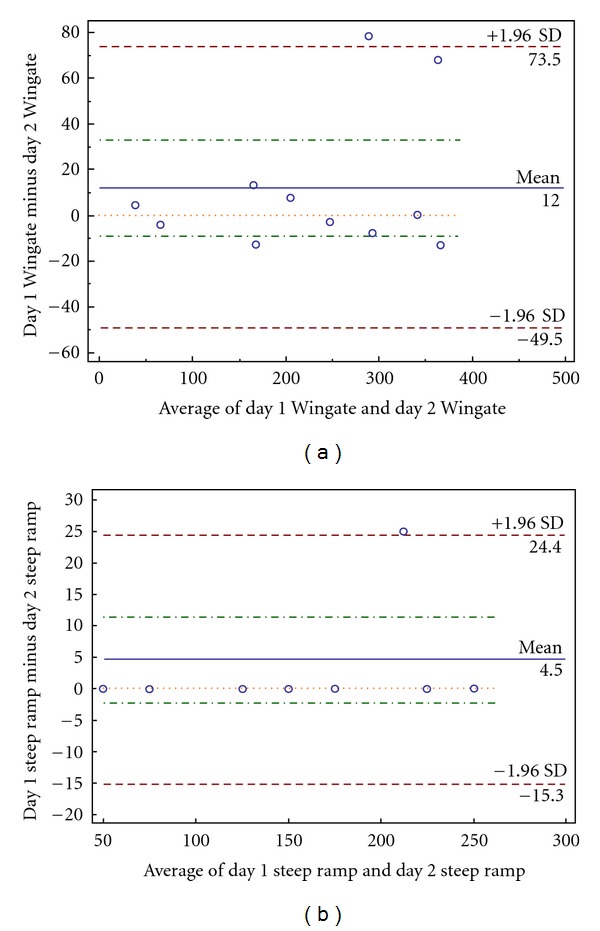
(a) Bland-Altman plot of reliability of Wingate average power measurements (W_avg_) between both sessions. *Y*-axis: The difference between W_avg_ from one day to the next. *X*-axis: The average of W_avg_ between both days. (b) Bland-Altman plot of reliability of the steep ramp peak power measurements (SR_peak_) between both sessions. *Y*-axis: The difference between SR_peak_ from one day to the next. *X*-axis: The average of SR_peak_ between both days.

Subject characteristics are presented in Table 1. Both the WAT and the SRAT demonstrated a high degree of test-retest reliability. ICC was 0.99 and 0.98, and the CV was 3.8% and 8.6% for the SRAT and WAT, respectively. Bland-Altman plots demonstrated a small degree of bias and error between the 2 sessions for the SRAT (4.5 W; −15.3–24.4 W, resp.) and the WAT (12.0 W; −49.5–73.5 W, resp.) (See Figures 1(a) and 1(b)).

**Figure 2:**
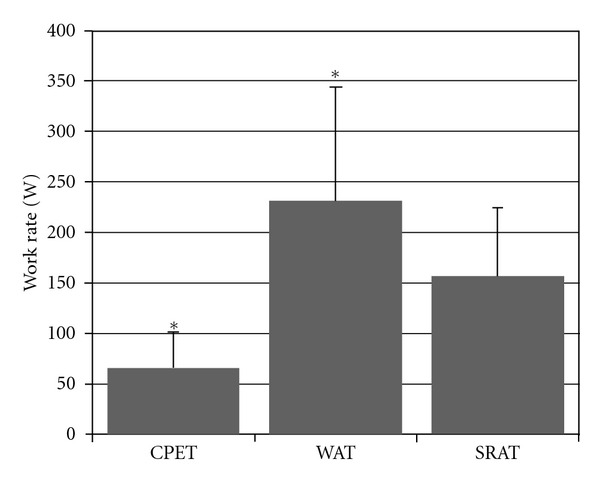
Comparison of the cardiopulmonary exercise test peak power (CPET), the steep ramp test (SRAT) peak power, and the average power in the 30-second Wingate anaerobic test (WAT) in watts. Results are presented as mean ±0.95 confidence interval. * = *P* < 0.05 versus SRAT.

Between-test physiological, ventilatory, and perceptual data are presented in Table 2. In addition, Figure 2 shows the mean work rates for the 3 tests. There were significant differences between CPET_peak_, SRAT_peak_, and W_avg_ (65.9 ± 35.6, 156.8 ± 67.9, and 231.2 ± 113.4 W, resp.). There were no differences between *V*_O_2__, RR, SpO_2_, HR, *V*_*E*_, IC, IRV, EELV, and *V*_*T*_/IC measurements at peak exercise in each of the 3 tests. *V*_CO_2__ at peak exercise (*V*_CO_2peak__) in the CPET was higher than *V*_CO_2peak__ in the WAT. *V*_CO_2peak__ in the SRAT was not significantly different from the other 2 tests. The respiratory exchange ratio (RER) at end exercise in the CPET was higher than both the SRAT and the WAT; however, the RER was not significantly different between the SRAT and the WAT. Dyspnea was significantly lower in the SRAT compared to the WAT; however, no difference in RPE in regards to leg fatigue between the tests.

Table 3 shows the correlation coefficients for the SRAT test data with respect to the corresponding data on each of the CPET and WAT tests. SRAT_peak_ correlated strongly with both the CPET_peak_ and the W_avg_. Most ventilatory and physiological parameters for the SRAT were found to correlate significantly with those on the CPET and WAT. Physiologic exercise performance variables tended to correlate better with the WAT, whereas ventilatory parameters tended to correlate better with the CPET.

## 4. Discussion

The primary purpose of this study was to determine the test-retest reliability of the SRAT. Our data demonstrate excellent retest consistency. All subjects but one obtained the same peak score on the SRAT between both test sessions. The reliability of the WAT was similarly assessed to determine the appropriateness of this test to be used as a criterion measure of anaerobic capacity in patients with COPD. Although reliability analysis of this test was not part of the purposes of this study, we demonstrated that the WAT was also a reliable measure. Reliability of the WAT has been previously established in health individuals [17] and patients with COPD using an abbreviated WAT [8]. Although the reliability of the SRAT has not yet been reported, incremental exercise tests of a smaller increment have demonstrated excellent reliability [18]; therefore, it is not surprising that the SRAT would also do so. The SRAT has been used in previous studies examining the effects of exercise training [9–11]; therefore, the results of the present study lend credibility to the use of the SRAT as an outcome measure in these previous, and future studies.

The secondary purpose of the present study was to compare the exercise responses and performance variables on the SRAT with those on the CPET and WAT. We demonstrated that the SRAT results in higher peak power output than the aerobic-based CPET, but lower than the anaerobic-based WAT. Despite these work load disparities, there were no differences in end-test oxygen consumption, heart rate, ventilation, and levels of ventilatory constraint between the tests. These findings complement those of Miyahara et al. [19] who demonstrated that, during CPET, higher ramp increments resulted in higher power outputs than lower ramp increments, despite similar cardiorespiratory responses; however, the ramp increment used in the SRAT was much higher than that used previously. As has been observed in patients with COPD during a CPET, limitations on the ability of patients to increase ventilation during exercise constrain performance, and consequently, oxygen consumption and heart rate [20]. Our study supports this assertion because we also found that mean values for peak heart rate were not maximal at end-exercise. The similar levels of metabolic demand and ventilatory limitation found in the present study suggest subjects performing any of the three tests are primarily limited by the inability to increase ventilation, rather than by a physiologically maximal oxygen consumption. Due to the short amount of time to complete the SRAT (67 ± 27 seconds) [21] and the high power output compared to the CPET, however, the SRAT elicits a greater degree of leg muscle anaerobic power than the CPET. In addition, the SRAT peak power was also strongly correlated with WAT average power output. These factors combined suggest that the SRAT may be a practical test of anaerobic power, even in the setting of ventilatory limitation.

Ventilatory constraint at end-exercise is suggested by an inability to further increase tidal volume due in part to dynamic hyperinflation [20] and by nearing predicted maximal ventilation. With dynamic hyperinflation, EELV increases, IRV decreases, and therefore *V*_*T*_ during exercise occupies a large percentage of IC [20]. In the present study, it was assumed that the patients would be limited by ventilatory factors during the CPET, in part due to reliance upon aerobic metabolism and the requirement to ventilate in proportion to aerobic demands. Therefore, it was also assumed that patients would hyperinflate less, demonstrate less ventilatory constraint (i.e., increased ventilatory reserve), and be limited more by peripheral muscle performance during tests lasting only 30–90 seconds (i.e., the SRAT). Despite the varying exercise durations, however, the similar level of ventilatory restriction observed at the end of the 3 tests suggests this may be a shared limiting factor in all of the tests. This common limitation may help to explain the high degree of correlation between the three tests.

The WAT_avg_ was significantly larger than the SRAT_peak_, and this may be partially related to the protocol design of the tests. The anaerobic metabolism present at the beginning of the WAT encourages high work rates without immediately driving ventilation. The patients gave maximal effort across the 30 seconds without realizing the degree of dyspnea they would incur due to the requirement for acid buffering, which was often near the end, or after cessation, of the WAT. Although not objectively measured in our study protocol, posttesting dyspnea scores were often reported to increase beyond the end-test values during immediate recovery from the WAT. In contrast, the SRAT, although also at a very high power output, builds incrementally to a patient-limited maximum. Patients were better able to control the amount of work performed prior to the development of disabling dyspnea, and the posttest increase observed in the WAT did not occur in the SRAT. For this reason, the SRAT seems to be an appropriate compromise between the low peak work rate of the CPET and the high work rate, but demanding recovery, of the WAT. The power output on the SRAT, albeit statistically lower than the WAT_avg_, combined with the short duration of the SRAT suggest that the SRAT reflects performance on an anaerobic power test (WAT), while allowing the patients to appropriately and safely manage their symptoms.

Since ventilation may have been a common limiting factor between the 3 tests, stratifying the population into categories of disease severity may have elicited different results in this study. Similarly, stratifying according to gender may have shown some differences. These options may be available in a study with a larger sample size.

This study demonstrates that the SRAT is a highly reliable measure of high-intensity muscle performance. In addition, it supports the assertion that leg power is often markedly underestimated in the traditional incremental design of the CPET, and that exercise is frequently terminated before a maximal muscular response has been achieved because of ventilatory limitations [6, 7, 22, 23]. Performance on the SRAT resulted in peak work rates 238% higher than that of the CPET. This is comparable to the findings of both Meyer et al. [10] and Puhan et al. [11], where SRAT_peak_ was approximately double the CPET_peak_ in chronic heart failure and COPD patients, respectively. Although further research is required, it is likely the SRAT would be more useful than the CPET in assessing and establishing intensities for exercise training that are sufficiently high to elicit clinical gains in leg muscle power and high-intensity performance.

## 5. Conclusions

The SRAT is a highly reliable, feasible, high-power test in patients with COPD and may be useful in estimating leg muscle power. CPET underestimates the capabilities of the leg muscles to perform high levels of work, due to the attainment of ventilatory limitations in COPD patients. Despite similar degrees of ventilatory constraint, the SRAT demonstrates markedly greater work rates and better reflects anaerobic performance in this population. The SRAT may thus be more suitable for prescribing high-intensity interval exercise in order to increase the potential for training benefit in COPD patients.

## Figures and Tables

**Table 1:** Subject characteristics.

Subject characteristics (*n* = 11)	
Male : Female, (*n*)	7 : 4
Age, years	71±3
Weight, kg	84.6 ± 21.0
BMI, kg/m^2^	29.3 ± 5.9
TLC, L (% predicted)	6.56 ± 1.21 (108 ± 10)
RV, L (% predicted)	3.42 ± 0.91 (151 ± 32)
FEV_1_, (L) (% predicted)	1.08 ± 0.26 (43 ± 15)
FVC (L), (% predicted)	2.73 ± 0.68 (83 ± 15)
FEV_1_/FVC, %	41 ± 10

Mean ± standard deviation. Abbreviations: TLC: total lung capacity, RV: residual volume, FEV_1_: forced expiratory volume in 1 second, FVC: forced vital capacity, pred = predicted.

**Table 2:** End-exercise measures for cardiopulmonary exercise test (CPET), steep ramp test (SR), and Wingate anaerobic test (WAT) presented with means and standard deviations.

End-exercise measures		Tests	
	CPET	SRAT	WAT
PWR (CPET & SR) Wavg (WAT)	65.9 ± 35.9	156.8 ± 67.9^∗†^	231.2 ± 113.4*
*V*_O_2__ (L/min)	1.11 ± 0.46	1.07 ± 0.41	0.99 ± 0.45
*V*_CO_2__ (L/min)	1.13 ± 0.52	0.97 ± 0.40	0.90 ± 0.42*
*V*_*E*_ (L/min)	40.436 ± 13.33	38.94 ± 13.01	39.73 ± 14.73
RER	1.00 ± 0.13	0.90 ± 0.07*	0.89 ± 0.08*
*V*_*T*_ (L)	1.19 ± 0.31	1.12 ± 0.24	1.09 ± 0.33
*V*_*T*_/IC (%)	76.5 ± 13.0	70.1 ± 12.0*	70.4 ± 13.8
IC/TLC (%)	24.1 ± 4.7	25.1 ± 5.5	23.5 ± 4.0
EELV/TLC (%)	75.9 ± 4.7	74.9 ± 5.5	76.5 ± 4.0
EILV/TLC (%)	94.0 ± 4.7	92.0 ± 5.1	92.9 ± 4.2
IRV/TLC (%)	6.0 ± 4.7	8.0 ± 5.1	7.1 ± 4.2
RR (breaths per minute)	34 ± 6	35 ± 8	37 ± 8
SpO_2_ (%)	91.5 ± 3.0	92.3 ± 1.5	93.3 ± 3.9
HR (beats per minute)	111.9 ± 20.9	109.8 ± 19.7	116.9 ± 22.0
HR (%pred)	75.3 ± 14.7	73.7 ± 13.0	78.5 ± 14.5
Dyspnea	5.6 ± 1.8	5.5 ± 2.1^†^	6.8 ± 2.3
Leg Fatigue	5.7 ± 1.7	5.6 ± 1.8	6.2 ± 1.9

Mean ± standard deviation. *: *P* < 0.05. ^†^indicates significance from WAT. PWR: peak work rate, *V*_O_2__: oxygen consumption, *V*_CO_2__: carbon dioxide elimination, *V*_*E*_: minute ventilation, RER: respiratory exchange ratio, *V*_*T*_: tidal volume, IC: inspiratory capacity, TLC: total lung capacity, EELV: end expiratory lung volume, EILV: end inspiratory lung volume, IRV: inspiratory reserve volume, RR: respiratory rate, SpO_2_: oxygen saturation, HR: heart rate.

**Table 3:** Pearson's *r* correlation coefficients between end-exercise measures during the SRAT and the cardiopulmonary exercise test (CPET) and Wingate anaerobic test (WAT).

End-exercise measures	Tests	
	CPET	WAT
PWR (CPET & SR) W_avg_ (WAT)	0.887*	0.887*
*V*_O_2__ (L/min)	0.891*	0.939*
*V*_CO_2__ (L/min)	0.837*	0.926*
*V*_*E*_ (L/min)	0.800*	0.930*
RER	0.549	0.615*
*V*_*T*_ (L)	0.907*	0.954*
*V*_*T*_/IC (%)	0.838*	0.806*
IC/TLC (%)	0.905*	0.873*
EELV/TLC (%)	0.905*	0.873*
EILV/TLC (%)	0.916*	0.880*
IRV/TLC (%)	0.916*	0.880*
RR (breaths per minute)	0.559	0.877*
SpO_2_ (%)	0.499	−0.017
HR (bpm)	0.684*	0.955*

*indicates significant correlation (*P* < 0.05).
